# Identification of Reference Genes in Human Myelomonocytic Cells for Gene Expression Studies in Altered Gravity

**DOI:** 10.1155/2015/363575

**Published:** 2015-01-13

**Authors:** Cora S. Thiel, Swantje Hauschild, Svantje Tauber, Katrin Paulsen, Christiane Raig, Arnold Raem, Josefine Biskup, Annett Gutewort, Eva Hürlimann, Felix Unverdorben, Isabell Buttron, Beatrice Lauber, Claudia Philpot, Hartwin Lier, Frank Engelmann, Liliana E. Layer, Oliver Ullrich

**Affiliations:** ^1^Institute of Anatomy, Faculty of Medicine, University of Zurich, Winterthurerstraße 190, 8057 Zurich, Switzerland; ^2^Department of Machine Design, Engineering Design and Product Development, Institute of Mechanical Engineering, Otto-von-Guericke-University Magdeburg, Universitätsplatz 2, 39106 Magdeburg, Germany; ^3^Study Group “Magdeburger Arbeitsgemeinschaft für Forschung unter Raumfahrt- und Schwerelosigkeitsbedingungen” (MARS), Otto-von-Guericke-University Magdeburg, Universitätsplatz 2, 39106 Magdeburg, Germany; ^4^Arrows Biomedical Deutschland GmbH, Center for Nanotechnology at the Westfälische Wilhelms-Universität Münster, Heisenbergstraße 11, 48149 Münster, Germany; ^5^German Aerospace Center, Space Agency, Königswinterer Straße 522-524, 53227 Bonn, Germany; ^6^KEK GmbH, Kemberger Straße 5, 06905 Bad Schmiedeberg, Germany; ^7^University of Applied Science Jena, Carl-Zeiss-Promenade 2, 07745 Jena, Germany; ^8^Zurich Center for Integrative Human Physiology (ZIHP), University of Zurich, Winterthurerstraße 190, 8057 Zurich, Switzerland

## Abstract

Gene expression studies are indispensable for investigation and elucidation of molecular mechanisms. For the process of normalization, reference genes (“housekeeping genes”) are essential to verify gene expression analysis. Thus, it is assumed that these reference genes demonstrate similar expression levels over all experimental conditions. However, common recommendations about reference genes were established during 1 g conditions and therefore their applicability in studies with altered gravity has not been demonstrated yet. The microarray technology is frequently used to generate expression profiles under defined conditions and to determine the relative difference in expression levels between two or more different states. In our study, we searched for potential reference genes with stable expression during different gravitational conditions (microgravity, normogravity, and hypergravity) which are additionally not altered in different hardware systems. We were able to identify eight genes (ALB, B4GALT6, GAPDH, HMBS, YWHAZ, ABCA5, ABCA9, and ABCC1) which demonstrated no altered gene expression levels in all tested conditions and therefore represent good candidates for the standardization of gene expression studies in altered gravity.

## 1. Introduction

Since several limiting factors for human health and performance in microgravity have been clearly identified [1], it has been concluded that substantial research and development activities are required in order to provide the basic information for appropriate integrated risk management, including efficient countermeasures and tailored life support systems [2]. In particular, bone loss during long stays in weightlessness still remains an unacceptable risk for long-term and interplanetary flights [3], and serious concerns arose whether spaceflight-associated immune system weakening ultimately precludes the expansion of human presence beyond Earth's orbit [4]. The immune and skeletal systems are tightly linked by cytokine and chemokine networks and direct cell-cell interactions [5, 6], and the immune system influences metabolic, structural, and functional changes in bones directly [6]. Both systems share common cellular players such as the osteoclasts, which are bone-resident macrophages and derivatives of monocytic cells. Therefore, knowing the cellular and molecular mechanisms of how gravity influences cell function is a valuable requirement to provide therapeutic or preventive targets for keeping important physiological systems fully functional during long-term space missions.

Since the first pioneering* in vitro* studies that revealed that cells of the immune system are sensitive to changes of gravitational force [7–10], several studies in real and simulated microgravity have confirmed microgravity-induced alterations in the molecular mechanisms and signal transduction processes in leukocytes, including the monocyte/macrophage system (MMS) [11, 12]. The MMS belongs to the innate immune system and is characterized by a fast but nonspecific immune reaction, the first line of defense against invading pathogens. Cells of the MMS in microgravity demonstrated disturbed cytokine release [13–15], reduced oxidative burst [16, 17], alteration of the cytoskeleton [18], and reduction in their locomotion ability [19]. Importantly, analysis of gene expression of monocytes during an ISS experiment revealed significant changes in gene expression associated with macrophageal differentiation [20].

Differential gene expression analyses are a widely used method to investigate the influence of different treatments or conditions on a cell system. The resulting changes on the molecular level can be investigated either by reverse transcription quantitative real-time PCR (RT-qPCR) as major technique for the sensitive and robust analysis of expression levels of specific genes [21–27] and microarrays for whole genome or transcriptome analyses [28].

After the genome sequencing era, when numerous genomes were completely decoded, the focus of interest shifted towards genome wide expression level analyses, so that a snapshot of the whole genome expression profile is obtained in a single experiment [28–30], offering also a possibility to obtain an insight into networks and pathways of biomolecular interactions on a large scale [29–33]. The technology behind microarray analysis developed fast, and different suppliers used different protocols for, for example, hybridization and data normalization. Therefore, it was and still is difficult to establish standards for the experimental procedure and processing of the raw data obtained [30]. Consequently, a concept for the development of standards for microarray experiments and data has been presented by the microarray gene expression database group (MGED) describing the minimum information about a microarray experiment (MIAME, [34]). This compilation covers (1) the experimental design, (2) the array design, (3) samples, (4) hybridizations, (5) measurements, and (6) normalization controls [34]. Also for RT-qPCR technique [35–38], standard guidelines (MIQE = minimum information for the publication of quantitative real-time PCR) were developed [27, 39–41].

One of the most crucial requirements of standardization are suitable internal controls so called reference genes that are used for data normalization, which are important to account for differences in the amount and quality of starting material as well as reaction efficiency [42]. GAPDH, HPRT, *β*-actin, tubulin, and ribosomal RNA genes are typical examples for frequently used reference genes [43–45]. However, reference genes have to be tested for their suitability as an endogenous control in each case prior to the experiment. This is of high importance substantiated by many studies reporting expression effects of classical “housekeeping genes” upon experimental treatments [46–49]. A selection of several reference genes used simultaneously can also be a good way to further increase reliability of the resulting data [50, 51]. In fact, recommendations state to identify three stable reference genes for each planned assay to assure a reliable outcome [50, 52]. The identification of stably expressed reference genes can be performed in a pilot study using dedicated algorithms like geNorm or BestKeeper or a combination hereof, where a minimum of eight potential reference genes are tested and ranked according to their stability being an indication for their suitability as control genes for normalization [50, 51]. Candidate reference genes for such a study may be, for example, chosen from the literature or from experimental data obtained from microarray analysis [27, 51].

However, common recommendations about reference genes were established during 1 g conditions and therefore their applicability in studies with altered gravity conditions has not been intensively demonstrated so far. Although, there are numerous publications describing differential gene expression analyses under simulated and real microgravity conditions in various cells types and tissues (supplementary Table 1 available online at http://dx.doi.org/10.1155/2014/363575), a systematic research on reference genes stable under altered gravity conditions has not been published yet.

In our study, we used microarray analyses to investigate the differential gene expression in U937 cells, a myelomonocytic human cell line, exposed to short-term (20 seconds) and middle-term (6 to 7 minutes) microgravity and hypergravity during parabolic flights and sounding rocket flights, two platforms commonly used by researchers to investigate the effects of real microgravity. Our experimental goal was to identify potential reference genes that can be recommended to the community of gravitational biology for differential expression analysis performed with cells of the immune system on those two frequently used platforms. Therefore, we chose 22 reference genes widely used throughout the literature and screened our microarray data for these particular genes evaluating their stability for possible application as control genes. Besides the highly conserved ribosomal RNA genes and others, ABC transporter and tRNA genes belong to evolutionary well-conserved genes as well. Since ribosomal RNA and tRNA genes are not represented on the array, we decided to adhere to tRNA related genes like tRNA synthetases, as these play a central role in basal cellular functions and should be robustly expressed to ensure cell survival. Therefore, our study comprised published reference genes, ABC transporters, and tRNA related genes. 

## 2. Material and Methods

### 2.1. Cell Culture

U937 cells (ATCC CRL1593.2) originating from a diffuse histiocytic lymphoma, displaying many monocytic characteristics, were used as a model cell line to investigate the differential gene expression under altered gravity conditions in monocytic/macrophageal cells. U937 cells were cultured in RPMI 1640 medium (Biochrom/Merck Millipore, Germany), supplemented with 10% fetal bovine serum (FBS Superior; Biochrom/Merck Millipore, Germany), 2 mM glutamine (Gibco/Life Technologies, Germany), and 100 U/mL penicillin as well as 100 *μ*g/mL streptomycin (Gibco/Life Technologies, Germany). Cells were seeded with a density of 0.2 × 10^6^ cells/mL and the medium was exchanged every 48 hours. Cells were harvested by centrifugation at 300 g for 5 min at room temperature, resuspended in fresh medium, and an aliquot was used for an adequate dilution with trypan blue to count the vital cell number. Cells were reseeded in fresh medium at a concentration of 0.2 × 10^6^ cells/mL.

### 2.2. Parabolic Flight Experiments

We designed and constructed an experiment module suitable to perform cell culture experiments with living mammalian cells during parabolic flights on board the Airbus A300 ZERO-G. During the 19th DLR parabolic flight campaign (PFC), we focused on the analysis of differential gene expression in U937 cells considering the different gravity conditions: in-flight 1 g, 1.8 g, and 0 g. Experiments were only performed during the first parabola to assure that the investigated differential gene expressions are generated by a direct effect of gravitational change and not an accumulated long-term effect. During the 19th DLR PFC, experiments were reproduced on two independent flight days.

In search of rapidly responsive molecular alterations in mammalian cells, short-term microgravity provided by parabolic flight maneuvers is an ideal instrument to elucidate initial and primary effects, without the influence and interference of secondary signal cascades. Parabolic flights provide 1 g, 1.8 g, and microgravity (*μ*g) with a quality of approximately 10^−2^ to 10^−3 ^g. For the 19th DLR PFC, 1 × 10^7^ U937 cells in 10 mL medium (RPMI 1640 supplemented with 100 U/mL penicillin, 100 *μ*g/mL streptomycin, 250 ng/mL amphotericin B (Gibco/Life Technologies, Germany), 2 mM glutamine, and 2% FBS (i.e., serum starved)) were filled into 200 mL Nutrimix bags (B. Braun Melsungen, Germany) and transported from the home laboratory to the preflight preparation laboratories at the NOVESPACE premises in Bordeaux, France. After arrival, cells were destarved by addition of 0.8 mL FBS per Nutrimix bag and used for the flight experiment on the following day. For the flight day, the Nutrimix bags were placed in a solid plastic container to create a double containment to prevent spillage of fluids in the aircraft in case of leakage which is strictly prohibited by the NOVESPACE regulations. The rapid preservation of the effects of altered gravity on the gene expression in the U937 cells was achieved by injection of 50 mL of RLT buffer (Qiagen, Germany), a lysis buffer immediately lysing cells and tissues prior to RNA isolation. The 1 g in-flight controls were performed 5 min before the first parabola and the 1.8 g sample directly before the microgravity phase of the first parabola. The *μ*g samples were fixed directly at the end of the microgravity phase of the first parabola. Samples were transported to the laboratory immediately after landing. 1 g ground controls were performed immediately after landing using the experimental module in the aircraft. In total, 30 samples were obtained during two parabolic flight days: 6x 1 g ground controls, 9x 1 g in-flight controls, 6x 1.8 g and 9x *μ*g.

### 2.3. RNA Isolation after the Parabolic Flight Experiments

After landing of the aircraft and transport of the samples to the laboratory on site facilities, the containers were disassembled, the Nutrimix bags were gently agitated, and the lysed cell solution was filled into a T75 straight neck cell culture flask. The cell solution was vortexed for 10 sec and passed four times through a Ø 0.8 × 120 mm needle (B. Braun Melsungen, Germany) fitted to a 50 mL syringe. 50 mL of absolute ethanol was added and precipitates were resuspended by vigorous shaking. A valve and a sterile connective piece were placed on a QIAvac 24 plus vacuum system (Qiagen, Germany) and an RNA maxi column (Qiagen, Germany) was attached to the connective piece. A vacuum of −200 mbar was adjusted, and the column was loaded with the lysed cell suspension. Then, the valve was closed, and the column was centrifuged at 4000 g for 3 min. 15 mL of buffer RW1 (Qiagen, Germany) was applied for washing membrane bound RNA. After centrifugation at 4000 g for 7 min, the flow through was discarded and two washing steps with 10 mL RPE buffer (Qiagen, Germany) followed with centrifugation at 4000 g for 3 min and 10 min, respectively. The column bound RNA was eluted by application of 600 *μ*L of RNase-free water (Qiagen, Germany), incubation for 1 min at room temperature, and centrifugation for 4 min at 4000 g. The elution step was repeated with the first eluate. The RNA was transported at approximately −150°C in a Cryo Express dry shipper (CX-100, Taylor-Wharton, USA) prepared with liquid nitrogen and stored at −80°C until the processing of the RNA for the microarray analysis.

### 2.4. Experiments during the TEXUS-49 Sounding Rocket Campaign

For the TEXUS-49 campaign at ESRANGE (European Space and Sounding Rocket Range, Kiruna, Sweden), U937 cells were cultured in the fully installed laboratories on site. Cells were seeded with a density of 0.2 × 10^6^ cells/mL and the medium was exchanged every 48 hours as described above. On the launch day, cells were visually inspected, harvested, counted, and pooled to a concentration of 5 × 10^7^ cells/mL. 0.5 mL of this cell suspension was filled in a sterile 3 mL plastic syringe shortly before the launch. Additionally, one syringe was filled with 0.3 mL of cell culture medium and another one with 1 mL Trizol LS (Life Technologies, Germany). The three syringes were mounted on a plastic block with a tubing system connecting them. This unit was finally integrated into the automatically operated experiment system. In total, 35 of these experiment units were prepared and were kept at 37°C until the integration into the payload of the rocket.

During the experimental run, firstly the 0.3 mL of medium, as a potential placeholder for an activation solution, and secondly the 1 mL of Trizol LS were injected to the cell suspension at defined time points to lyse the cells and preserve the current status of differential gene expression. This sequential injection of fluids was performed at 75 sec after launch to monitor the so-called baseline (BL) directly before the *μ*g phase and at 375 sec after launch shortly before the end of the *μ*g phase. A group of 1 g ground controls were kept on ground in the incubator simultaneously to the *μ*g sample group.

TEXUS-49 consisted of a VSB-30 engine (S-30 solid rocket stage with an S-31 second stage) and of the payload. The rocket was launched on March 29, 2011 at 06:01 a.m. from the ESRANGE Space Center near Kiruna, Sweden. During the ballistic suborbital flight, an altitude of 268 km and 378 sec of microgravity with a quality of 10^−5 ^g were achieved. Further parameters include first stage peak thrust acceleration 6.3 g, mean thrust acceleration 5.03 g, burnout at 12.3 sec, and engine separation at 13.6 sec; second stage peak thrust acceleration 13.5 g, mean thrust acceleration 7.30 g, burnout at 43.0 sec, yo-yo despin at 56.0 sec, and engine separation at 59.0 sec.

### 2.5. RNA Isolation after the TEXUS-49 Sounding Rocket Campaign

Directly after landing, localization, and recovery of the payload, the experiment modules were dismantled and handed over to the scientists. The cell suspension was sheared three times with a 20 G needle (B. Braun Melsungen, Germany) and distributed in two 2.0 mL tubes. 0.1 mL of chloroform (Sigma-Aldrich, Germany) was added, and the homogenate was vortexed for 15 sec and incubated for 5 min at room temperature before a 15 min centrifugation step at 11000 g and 4°C. The upper phase of both 2.0 mL tubes was transferred into a 15 mL tube, and 4 mL of RLT buffer as well as 3 mL of absolute ethanol was added and mixed. 4 mL of this solution was pipetted on an RNA Midi column (Qiagen, Germany) and centrifuged for 30 sec at 3000 g and room temperature. The flow through was discarded and the residual 4 mL of RNA solution was loaded on the column and centrifuged for 5 min at 3000 g at room temperature. Then, the columns were washed twice with 2.5 mL of RPE buffer and centrifuged for 2 min and 5 min, respectively, at 3000 g at room temperature. The RNA was eluted by addition of 250 *μ*L RNase-free water (Qiagen, Germany) to the column, incubation for 1 min at room temperature, and centrifugation for 3 min at 3000 g and room temperature. The eluate was loaded again onto the column, followed by a 1 min incubation and centrifugation for 5 min at 3000 g and room temperature. The isolated RNA was transferred into sterile cryotubes and stored until the return transport at approximately −150°C in a Cryo Express dry shipper (CX-100, Taylor-Wharton, USA) prepared with liquid nitrogen. After arrival in the home laboratory, samples were stored at −80°C until the processing of the RNA for the microarray analysis.

### 2.6. RNA Processing and Microarray Analysis

RNA quantity and purity were analyzed spectrophotometrically using a NanoDrop 1000 (Thermo Scientific, USA). Isolated RNA samples were all of high quality with 260/280 nm ratios between 1.9 and 2.1. The RNA integrity was measured using an Agilent 2100 Bioanalyzer (Agilent Technologies, USA). Only RNA with an RNA integrity number (RIN) > 8.7 was used for the following microarray analysis. 400 ng total RNA was applied to Cy3-labeling with the “Low RNA Input Linear Amplification Kit, PLUS, One-Color” (Agilent Technologies) and hybridized for 17.5 h to a NimbleGen expression microarray (12 × 135,000 features) employing the “Gene Expression Hybridization Kit” (Agilent Technologies). Afterwards, arrays were washed and scanned by the Microarray Scanner G2505B (Agilent Technologies).

The image files of the scanner were analyzed with the NimbleScan Software 2.6 using the robust multiarray analysis (RMA) with the default parameters. RMA, a probe-level summarization method, identifies probes that are outliers in the overall behavior of the expression measured for a given gene. The contribution of outlier probes is reduced in the reported gene expression level, which has been demonstrated to improve the sensitivity and reproducibility of microarray results. In addition to screening outlier probes, NimbleScan software's implementation of RMA [53] used quantile normalization and background correction.

The normalized microarray data were analyzed using Partek Genomics Suite 6.6. Statistical analysis was performed using the one-way ANOVA and the false discovery rate (FDR) [54] for multiple-testing correction. Further, the coefficient of variation (CV) expressed in percent was calculated, also known as “relative variability.” It equals the standard deviation divided by the mean. An integration tool (available at http://www.leonxie.com/referencegene.php) [50, 51, 55] of four algorithms (geNorm, NormFinder, BestKeeper, and the comparative delta-CT method) was used to evaluate the expression stability of the reference genes. On the basis of the resulted rankings from the four algorithms, an overall ranking of the candidate genes was achieved.

### 2.7. Statistical Analysis of Selected Genes

Genes of interest were identified, and the log 2 values of the measured fluorescent intensities returned by the Partek software were back calculated to linear values. Then, means of all values of the same gene generated by different probes were calculated, if at least three values existed excluding outliers. Subsequently, standard deviations were calculated for the means and an unpaired *t*-test with Welch correction was performed using Excel 2011 (*t*-test, tails 2, type 3) to obtain statistical significance.

## 3. Results

The aim of our study was to identify a group of genes that show a stable, nonchanging expression profile in immune cells under altered gravity conditions over a time range of seconds until several minutes. Therefore, we performed experiments on the 19th DLR PFC and the sounding rocket mission TEXUS-49, two platforms that offer microgravity times of 20 seconds and 6 minutes, respectively. During both missions, U937 cells, a model for monocytic/macrophageal cells of the human immune system, were exposed to different gravity conditions for various time periods (see Table 1). During the 19th DLR PFC, cells were exposed only to the first parabola with the following sequence: 1 g in-flight control, 1.8 g, and microgravity (*μ*g). Cells were subjected to altered gravity conditions of 1.8 g and *μ*g for 20 seconds in each case and were immediately fixed and stored cooled until RNA isolation. In case of the TEXUS-49 campaign, cells underwent the following sequence of altered gravity: hypergravity up to 13.5 g during the first 75 seconds after liftoff and *μ*g for 378 seconds. Hypergravity is defined as the baseline (BL), because samples mirror the vibration and hypergravity effects directly before the microgravity phase. In both experimental setups, on ground 1 g hardware controls (H/W) were performed to be able to differentiate between the effects caused by the conditions experienced before hypergravity and *μ*g and the altered gravity conditions themselves. After the campaigns, the RNA samples were analyzed for quantity and quality by NanoDrop spectrophotometry and a bioanalyzer analysis, and only samples with an RNA integrity number (RIN) higher than 8.7 were chosen for subsequent microarray analysis. 12 × 135 K Roche NimbleGen arrays were hybridized and data were collected after the normalization procedure. In total, we obtained data from 46 single microarrays (19th DLR PFC: 8x *μ*g, 6x H/W, 8x 1 g, and 6x 1.8 g; TEXUS-49: 7x *μ*g, 6x H/W, and 5x BL).

**Figure 1:**
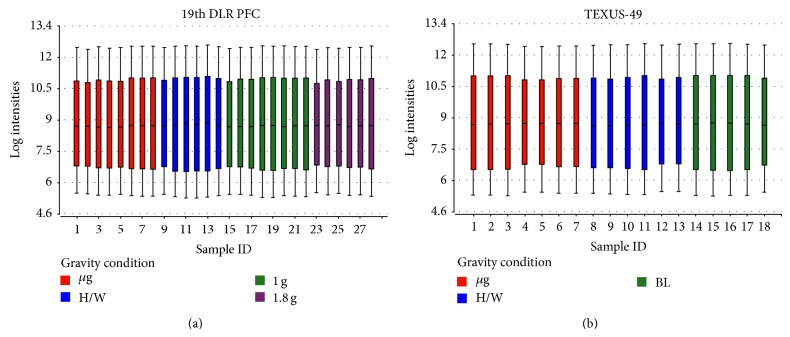
Boxplots showing the log expression values of individual microarrays. The central line represents the 50th percentile or median, whereas the upper and lower boundaries of the box display the 75th and 25th percentile, respectively. The upper and lower bars represent the 9th and the 91st percentile. Two experimental data sets are displayed, (a) 28 microarrays hybridized with samples from the 19th DLR PFC (8x *μ*g, 6x H/W, 8x 1 g, 6x 1.8 g) and (b) 18 microarrays hybridized with samples originating from the TEXUS-49 campaign (7x *μ*g, 6x H/W, 5x 1 g). The expression data show an even distribution for the displayed log intensities.

Data tables were compiled individually for the 19th DLR PFC and TEXUS-49 including all gravity conditions listed in Table 1, and a first overview of the datasets was provided by a boxplot diagram (Figures 1(a) and 1(b)). Boxplots are a useful tool to visualize the variation within a microarray and between microarrays. The central line shows the position of the median, while the upper and the lower boundaries represent the upper (75th percentile) and lower (25th percentile) quartile. The ends of the tails display the 9th and the 91st percentile. The boxplots of the microarray data show that there is only little variation within a single array and between the arrays that belong to the same gravity condition.  Figure 1 shows that the quality of both data sets (19th DLR PFC and TEXUS-49) is sufficient to proceed with further analyses.

**Figure 2:**
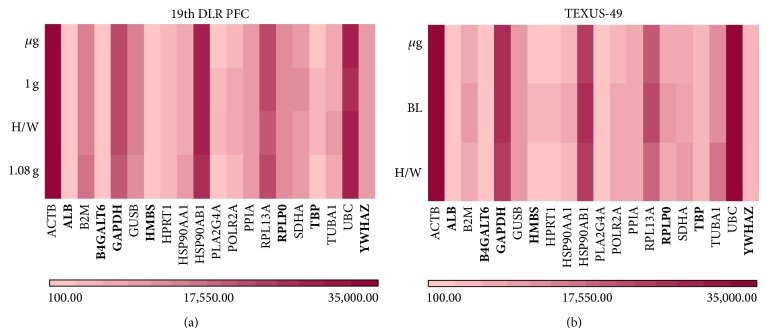
Heatmaps for selected reference genes. The graph illustrates fluorescent intensity levels of the 20 potential reference genes from Table 2 between the three and four different gravity conditions, respectively. Each gene is represented in one column, and each gravity condition is represented in one row. (a) *μ*g, H/W, 1 g, and 1.8 g (19th DLR PFC) and (b) H/W, BL, and *μ*g (TEXUS-49). The heatmap shows large variation in fluorescence intensities for the different genes. However, within the same gene, expression levels are similar for all tested conditions. The lower bar with the graduated red colors is the measure for the different fluorescence intensities.

**Figure 3:**
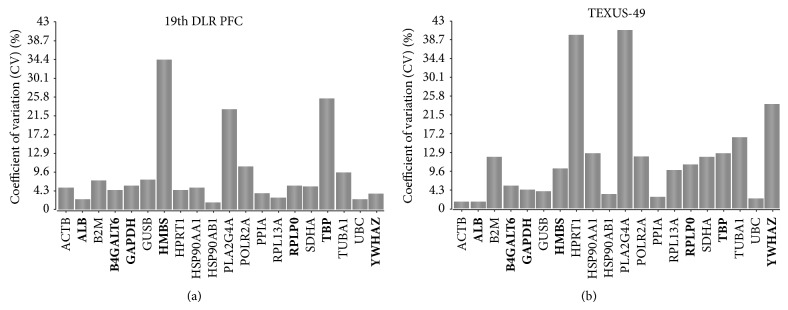
Coefficient of variation calculation for the potential reference genes. This bar chart displays the coefficient of variation (CV) in % of the 20 potential reference genes across the gravity conditions for the 19th DLR PFC (H/W, 1 g, 1.8 g, *μ*g) and TEXUS-49 (H/W, BL, *μ*g). A lower value corresponds to higher stability in gene expression. (a) 19th DLR PFC: All calculated CV values are below the threshold of 50%. (b) TEXUS-49: all CV values are below 50%, but in total more genes show higher coefficients of variation.

In search of potential reference genes for gravitational studies in this monocytic/macrophageal cell system, we first performed PubMed database search to identify commonly used reference genes in RNA expression analyses in human cells. We found 22 genes that were used in several reverse transcription quantitative real-time PCR (RT-qPCR) studies as control genes for normalization (Table 2, supplementary Table 2). The microarray data tables were screened for these 22 widely used reference genes, and 20 of them could be located on the Roche NimbleGen 12 × 135 K array that was used in our experiments. Two genes coding for 5s and 18s rRNAs could not be identified, since they are not spotted on the array. The PFC and TEXUS data sets were screened for those 20 selected potential reference genes, and fluorescence intensities were compiled for each gene and each gravity condition in heatmaps (Figures 2(a) and 2(b)). Overall fluorescence intensities for all samples showed only minor differences in the heatmaps. A more detailed visual inspection revealed completely equal fluorescence intensities for ACTB, ALB, B4GALT6, HMBS, HPRT1, PPIA, RPLP0, and YWHAZ for the gravity conditions prevailing during the 19th DLR PFC (Figure 2(a)). The gravity conditions investigated during the TEXUS-49 campaign showed stable expression values for the genes ACTB, ALB, B4GALT6, GUSB, PLA2G4A, POLR2A, PPIA, TBP, UBC, and YWHAZ (Figure 2(b)). For further characterization and identification of stable reference genes, we performed a geNorm pilot study [51] and calculated the coefficient of variation (CV) for all 20 potential reference genes (Figure 3). For homogeneous groups, CV values below 25%, and for heterogeneous groups, CV values below 50% are acceptable [56]. Rapid and extreme changes in gravity induce strong changes in cellular functions. Therefore, we classified our samples as heterogeneous groups. According to the set criteria, all analyzed potential reference genes showed CV values below 50% for the PFC and TEXUS data sets (Figures 3(a) and 3(b)). In the sample set of the 19th DLR PFC, all genes but HMBS fulfill even the more stringent criterion of a CV below 25% (Figure 3(a)). For the samples collected during the TEXUS-49 campaign, all genes but HPRT1 and PLA2G4A display CV values below 25% (Figure 3(b)).

**Figure 4:**
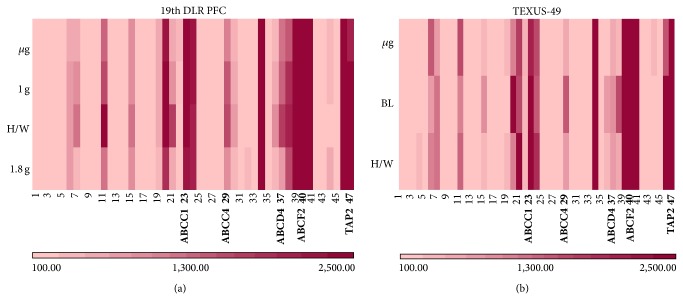
Heatmaps for highly conserved ABC transporters. The fluorescent intensity levels of the 47 ABC transporter genes shown in supplementary Table 2 were quantified for the different gravity conditions. Each gene is represented in one column, and each gravity condition is represented in one row. (a) *μ*g, 1 g, H/W, and 1.8 g (19th DLR PFC) and (b) *μ*g, BL, and H/W (TEXUS-49). The heatmaps show large variation in fluorescence intensities for the different genes. However, within the same gene, expression levels are mostly similar for all tested conditions. The lower bar with the graduated red colors is the measure for the different fluorescence intensities.

**Figure 5:**
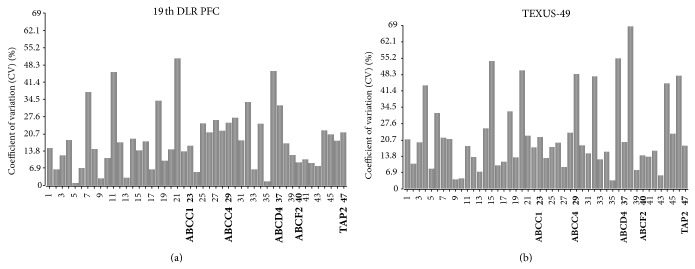
Coefficient of variation calculation for the ABC transporter genes. This bar chart displays the coefficient of variation (CV) in % of the 47 ABC transporter genes across the gravity conditions for the 19th DLR PFC (H/W, 1 g, 1.8 g, *μ*g) and TEXUS-49 (H/W, BL, *μ*g). A lower value corresponds to higher stability in gene expression. (a) 19th DLR PFC: all calculated CV values are below the threshold of 50% and fulfill the criterion. (b) TEXUS-49: three genes show CV values higher than 50% and were excluded from further analyses. The numbers correspond to the ABC transporters listed in supplementary Table 2. Genes that were further analyzed are labeled and marked in bold (ABCC1, ABCC4, ABCD4, ABCF2, and TAP2).

To increase the number of potential reference genes that can be used as standards for differential expression analyses in gravitational studies, we extended our analysis to evolutionary highly conserved genes. We hypothesized that genes stable over time and taxonomic kingdoms should have very fundamental functions within a cell and thus be largely independent from external influences to ensure basic cellular functions. Besides ribosomal RNA genes, which are not represented on the microarray applied in this study, ABC transporters and tRNA genes are also evolutionary highly conserved over a wide variety of organisms. Unfortunately, the 12 × 135 K Roche NimbleGen array does also not contain probes for tRNA. Therefore, we had a look at expression profiles of ABC transporters and tRNA related genes (supplementary Tables 2 and 3). Since almost all fluorescence values of tRNA related genes showed a high variance making reasonable analysis impossible, we concentrated on the ABC transporters. Heatmap analyses were carried out to obtain a first impression on the gene stability (Figure 4). The samples from the 19th DLR PFC and TEXUS-49 mission also show a rather high variation in fluorescence intensities (Figures 4(a) and 4(b)). The calculation of the CV for these samples (Figure 5) displays higher values compared to the potential reference genes; however, taken together all analyzed samples of the 19th DLR PFC fulfill the criterion of CV values less than 50% in case of ABC transporter signals (Figure 5(a)). Out of 47 samples, 36 are also below 25% CV. Although three samples from TEXUS-49 showed values above 50% CV (Figure 5(b)), 37 samples stayed below the 25% threshold (Figure 5(b)). Selected reference genes and ABC transporters (marked in bold, Figures 4 and 5) were chosen for further detailed analysis of differential gene expression under altered gravity conditions.

**Figure 6:**
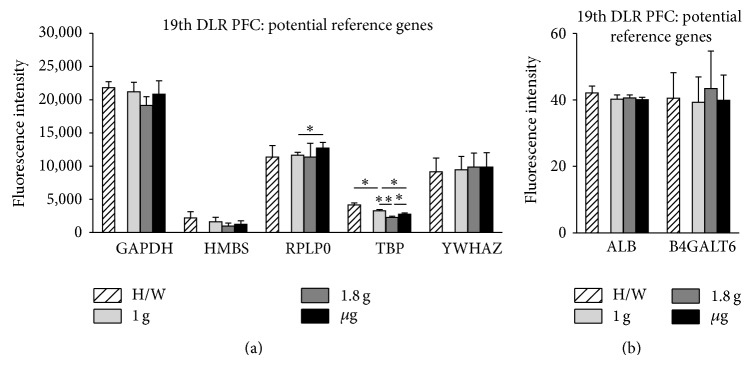
Influence of altered gravity during parabolic flight on potential reference genes. RNA expression levels after 1 g (light gray), 1.8 g (dark gray), and *μ*g (black) conditions during the 19th DLR parabolic flight campaign. Hardware ground controls (H/W, striped) are shown for each experimental group. RNA expression levels are shown as fluorescence intensities. (a) The expression values for GAPDH, HMBS, RPLP0, TBP, and YWHAZ are displayed. (b) ALB and B4GALT6 show low but stable fluorescent intensities. GAPDH, HMBS, YWHAZ, ALB, and B4GALT6 show no significant change in RNA levels upon altered gravity for 20 sec, while RPLP0 displays *μ*g sensitivity compared to 1 g and TBP reacts sensitively to all g conditions. Mean values of at least three measurements with standard deviations are shown. ^*^ *P* < 0.05, ^**^ *P* < 0.005.

**Figure 7:**
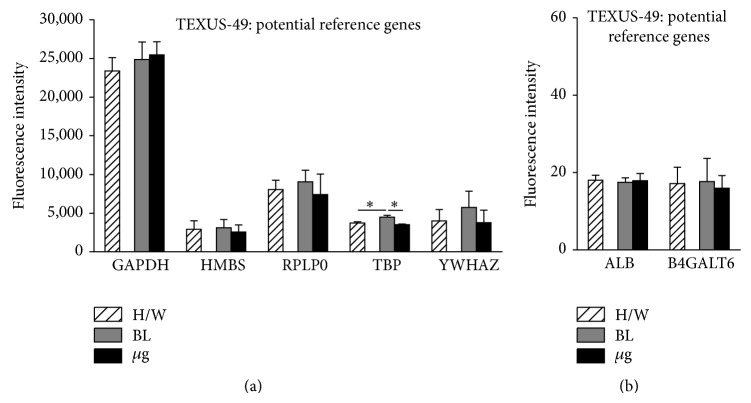
Influence of altered gravity during sounding rocket flight on potential reference genes. GAPDH, HMBS, RPLP0, TBP, and YWHAZ (a), ALB and B4GALT6 (b) RNA expression levels after launch and acceleration (BL, dark gray) and *μ*g (black) conditions of TEXUS-49. Hardware ground controls (H/W, striped) are shown for each experimental group. RNA levels are displayed as fluorescence intensities. GAPDH, HMBS, RPLP0, YWHAZ, ALB, and B4GALT6 show no significant change in RNA levels upon altered gravity, while TBP reacts sensitively to all g conditions. Mean values of at least three measurements with standard deviations are shown. ^*^ *P* < 0.05.

For nine of the potential reference genes from the literature, there were at least three values returned by the microarray, generated by independent probes targeting the same gene. Two of these genes were excluded from further analysis due to high variance between their single values (HSP90AA1 and PPIA), and the remaining seven genes (ALB, B4GALT6, GAPDH, HMBS, RPLP0, TBP, and YWHAZ (see Table 3)) were subjected to further statistics. The calculation of the mean fluorescence intensity levels revealed that different ranges of transcript abundance are present in both experimental setups. While ALB and B4GALT6 seem to be expressed rather low, GAPDH, HMBS, RPLP0, TBP, and YWHAZ are represented in much higher abundance (Figures 6 and 7). The comparison of mean fluorescence intensities of one gene under different g conditions revealed that GAPDH, HMBS, YWHAZ, ALB, and B4GALT6 are stably expressed with respect to all investigated gravity conditions during parabolic flight of the 19th DLR PFC (Figures 6(a) and 6(b)). RPLP0 is significantly upregulated by *μ*g compared to 1 g, while TBP is initially downregulated by 1.8 g and recovers during *μ*g (Figure 6(a)). Furthermore, comparison of in-flight 1 g controls to 1 g ground controls (H/W) shows a significantly reduced mRNA level of TBP portending that during the preexperimental phase a certain kind of stress was accumulated in the cells influencing its expression level.

The data analysis of the TEXUS-49 sounding rocket experiment reveals stable RNA expression levels throughout the different g levels for GAPDH, HMBS, RPLP0, YWHAZ, ALB, and B4GALT6 (Figures 7(a) and 7(b); Table 4, and supplementary Table 1). TBP RNA levels were reduced in *μ*g samples compared to the in-flight BL. Interestingly, comparisons between the H/W ground controls and BL revealed a postlaunch increase in RNA expression most likely induced by the launch vibrations or hypergravity (Figure 7(a)).

Only a very low number of tRNA related genes fulfilled our criterion of being represented by at least three probes (four out of 32). For three out of those four genes, fluorescent intensity showed a great variance between the single values as mentioned above. Only one tRNA synthetase (SARS) yielded reasonable results. The exposure of the cells to altered gravity conditions during the parabolic flight resulted in a decreased SARS expression in 1 g in-flight control and 1.8 g samples compared to the H/W ground control and 1 g control, respectively (supplementary Table 3). Although not significant, there is a visible increase of SARS mRNA upon *μ*g compared to 1.8 g arguing for an immediate expression recovery after termination of 1.8 g. This is in line with the results from TEXUS-49 flight campaign where SARS shows no significant expression change in in-flight baseline control or in *μ*g compared to H/W ground control. This could be due to fast expression recovery of the gene during g alterations, hyper-g phase, and *μ*g.

**Figure 8:**
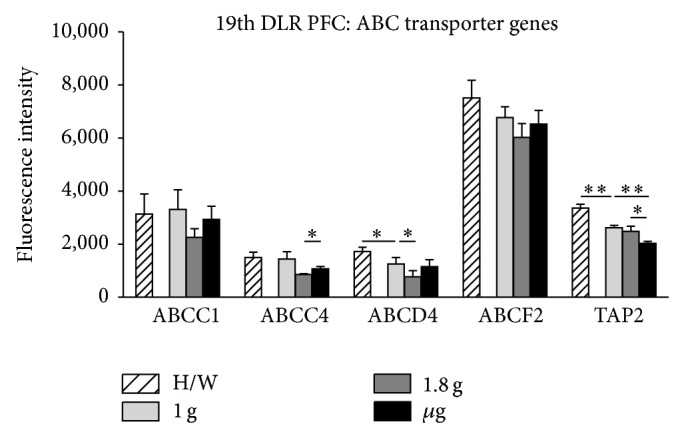
Influence of altered gravity during parabolic flight on ABC transporter genes. ABCC1, ABCC4, ABCD4, ABCF2, and TAP2 RNA levels after 1 g (light gray), 1.8 g (dark gray), and *μ*g (black) conditions during the 19th DLR parabolic flight campaign. Hardware ground controls (H/W, striped) are shown for each experimental group. RNA expression levels are displayed as fluorescence intensities. ABCC1 and ABCF2 show no significant change in RNA expression levels upon altered gravity, while ABCC4 and TAP2 display *μ*g sensitivity compared to 1.8 g and to 1.8 g and 1 g, respectively. ABCD4 reacts sensitively to 1.8 g compared to 1 g, and ABCD4 and TAP2 show vibration sensitivity comparing 1 g to H/W. Mean values of at least three measurements with standard deviations are shown. ^*^ *P* < 0.05, ^**^ *P* < 0.005.

**Figure 9:**
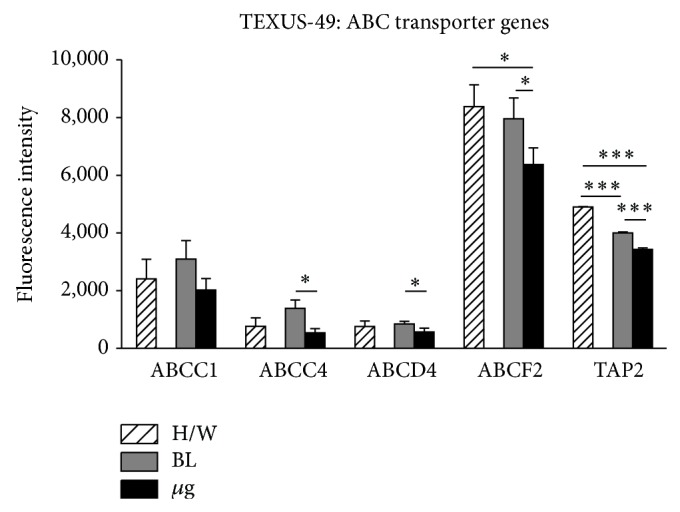
Influence of altered gravity during sounding rocket flight on ABC transporter genes. ABCC1, ABCC4, ABCD4, ABCF2, and TAP2 RNA expression levels after launch and acceleration (BL, dark gray) and *μ*g (black) conditions of TEXUS-49. Hardware ground controls (H/W, striped) are shown for each experimental group. RNA levels are depicted as fluorescence intensities. Only ABCC1 expression is stable over all g conditions. ABCC4, ABCD4, ABCF2, and TAP2 display *μ*g sensitivity compared to BL and to H/W in the case of ABCF2 and TAP2. TAP2 also shows vibration sensitivity comparing BL to H/W. Mean values of at least three measurements with standard deviations are shown. ^*^ *P* < 0.05, ^**^ *P* < 0.005, and ^***^ *P* < 0.0005.

The highly conserved ABC transporters were represented as a large group of genes on the applied microarray. We analyzed a total of 47 ABC transporters belonging to nine different sub-families (supplementary Table 2). 19 ABC transporter genes were represented by three or more individual probes on the microarray and 11 of them had similar fluorescent intensities meeting the requirements for a statistical analysis. Exemplarily, five of those 11 ABC transporters are shown in Figures 8 and 9.

During the short-term gravity alterations achieved by parabolic flights, ABCC1 and ABCF2 displayed no significant differential expression between all g conditions analyzed. TAP2 showed a significant reduction of RNA expression comparing *μ*g samples to 1.8 g samples, while ABCC4 showed an increase. ABCD4 revealed hyper-g sensitivity by reducing its RNA level during 1.8 g compared to 1 g. And ABCD4 and TAP2 displayed reduced expression during preflight phase compared to H/W control (Figure 8, Table 5).

A prolonged exposure of the cells to *μ*g (378 sec versus 20 sec) during TEXUS-49 experiment led to significant reduction of mRNA levels of ABCC4, ABCD4, ABCF2, and TAP2 in *μ*g compared to in-flight BL. Furthermore, TAP2 expression already decreases in the first phase after launch (BL versus H/W), while the other ABC transporters' mRNA levels appeared stable (Figure 9, Table 6).

Taken together, in this study, we identified eight genes as nonchanging reference genes suitable for studies under altered gravity conditions, and nine genes as candidates for g-sensitivity and 83 genes could not be assigned to either group due to low probe number on the microarray or to great variance between the probe values (Table 7).

## 4. Discussion

Microarray expression data are intensely used to analyze differential gene expression in cells, tissues, and organisms that are exposed to various conditions [29, 30]. Even in the field of gravitational biology, gene expression analyses are utilized with increasing frequency. Recently, an article was released giving an overview of all published microarray based microgravity studies [57] describing the difficulties to combine and overlay the data from different experiments, study objects, microgravity platforms (simulated microgravity, sounding rocket, space shuttle, and ISS missions), and different microarray experimental designs [58–64]. The different analyses were done mostly in simulated microgravity and investigated various organisms and cell types like* Arabidopsis thaliana, Salmonella enterica*, and rat and mouse tissues, as well as human osteoblasts and T-cells [58–61, 65–70]** (**for the complete list see [57]). The goal was to screen the vast amount of data to identify a list of major “space genes” that are sensitive to microgravity throughout all involved platforms. The data inspection revealed a huge number of differentially expressed genes but with only little or no overlap between closely related studies on the level of single genes. In contrast, on the level of pathway analysis, it was possible to define major pathways like ECM-receptor interaction, focal adhesion, TGF-beta signaling, and glycolysis being affected in many species (human, mouse, rat, and* Xenopus*, in different combinations) by the exposure to microgravity [57]. Moreover, major “space genes” sensitive to microgravity were defined, if they were found to be differentially expressed in at least four of the examined studies. The results showed in total eight potential space genes (CD44, MARCKS, FN1, TUBA1, CTGF, CYR61, MT2, and MT1), which are involved in T-cell development, cell motility, extracellular matrix components, cytoskeleton, and oxidative stress protection [57]. The study describes in detail the difficulties of combining gene expression data from different groups due to varying experimental setups and conditions. It elucidates that it is of high relevance to be able to standardize gene expression data that arose from RT-qPCR or microarray studies. A key component for standardization within a single experiment and between different experiments is normalization. An important factor for normalization is the use of stable reference genes. There are numerous studies describing that commonly used reference genes could represent a pitfall, because they are often differentially expressed under specific experimental conditions and that they have to be considered carefully before the experiment [46–49]. Different guidelines have been published to facilitate standardized experimental design and increase comparability between analyses (MIQE, MIAME) [34, 39, 40]. It is, for example, highly recommended to perform a pilot study with programs like geNorm or BestKeeper prior to the experiment to identify several stable reference genes that can be used simultaneously as controls for normalization in the differential gene expression analysis [50, 51].

Alternatively, to microarrays a novel technique, RNA-Seq, is under development for whole genome expression analyses. It is reported that this method has advantages in detecting low abundance transcripts, genetic variants, and splice isoforms of genes as well as distinguishing biologically critical isoforms [71]. Despite the described technical advantages of RNA-Seq, microarrays remain popular for some reasons. The microarray platforms have a proven track record spanning nearly two decades in the lab. The arrays are generally considered easier to use with less complicated and less labor-intensive sample preparation than RNA-Seq. The same holds true for the data storage and data analysis. Moreover, despite the rapid drop in the cost associated with next-generation sequencing (NGS), arrays are still more economical and yield higher throughput, providing significant advantages when working with a large number of samples. Therefore, microarray analyses are still more commonly used for transcriptional profiling experiments [71].

Taking into account that many other studies throughout the last few years have reported a considerable portion of the traditionally used reference genes not being stably expressed under various experimental conditions, it becomes rather apparent that a natural constant as 1 g might have even more an effect on the expression of genes than other test circumstances. Therefore, we focused in this study on the investigation of the expression qualities of several potential reference genes under 1 g compared to altered gravity conditions generated by two widely used platforms: parabolic flights and sounding rockets. These two platforms are of special interest because of the rather easy access compared to the extremely limited accessibilities of long-term microgravity experiments on satellites and the ISS. We present a microarray based analysis identifying stable reference genes in cells of the immune system exposed to short-term (several seconds) and middle-term (several minutes) altered gravity conditions on the two widely used platforms: parabolic flights and sounding rockets.

Our analyses of commonly used reference genes, ABC transporters and tRNA related genes, revealed that nine of the 17 genes suspected to be ubiquitously expressed are g-sensitive and therefore inappropriate for our purposes, amongst them being TATA box binding protein (TBP), a fundamental transcription factor for many genes, and seryl-tRNA synthetase (SARS), an essential enzyme for mRNA translation, also regulating vascular development (Table 7).

Two of the g-sensitive genes, that we identified in this study, are involved in multidrug resistance processes like the ABC transporters ABCC4 and transporter associated with antigen presenting 2 (TAP2). ABCC4 is of particular interest, because it has the ability to provide resistance to antiviral and anticancer nucleotide analogs and methotrexate [72, 73], acts as an independent regulator of intracellular cAMP, mediates cAMP dependent signal transduction to the nucleus, and controls human and rat smooth muscle cell (SMC) proliferation [74]. It is known that cAMP has largely inhibitory effects on components of macrophage activation and elevation of cAMP levels which suppresses FcgammaR-mediated phagocytosis [75]. Therefore, it would be interesting to look at this multidrug resistance-associated protein (ABCC4) in more detail in microgravity exposed cells to elucidate its role in the signaling cascades important for immune cell action and reaction under space conditions, as ABCC4 proved to be *μ*g-sensitive during parabolic and sounding rocket flight (Figures 8 and 9). Furthermore, TAP2 seems to be even more g-sensitive because it shows significant differential gene expression under *μ*g and hypergravity conditions during parabolic and sounding rocket flight (Figures 8 and 9). It will be interesting to further analyze the potential effects of differential gene expression of TAP2 because it is a key player in endogenous pathways for antigen presentation and involved in the cellular transport of antigens for subsequent association with MHC class I molecules [76]. An imbalance in its gene expression could lead to an impaired reactivity of cells of the immune system under altered gravity conditions.

Further standard genes as well as ABC transporters like RPLP0, ABCD4, and ABCF2 also turned out in our analysis to be g-sensitive. RPLP0 encodes for a ribosomal protein that is a component of the 60S subunit and interacts with P1 and P2 to form pentameric complexes [77]. It is involved, for example, in Chagas disease [78] as well as mixed connective tissue disease [79]. The ABC transporters ABCD4 and ABCF2 are involved in transport of molecules across extra- and intracellular membranes like in peroxisomal import of fatty acids and/or fatty acyl-CoAs in the organelle [80] and play a role in suppression of volume-sensitive outwardly rectifying Cl channel (VSOR), respectively [81]. Altered expression levels of those genes by microgravity or hypergravity could have an impact on the translational level or the supply of the cell with essential resources important for proper cellular function. Recently it was shown that during parabolic flights the activity of the MRP2-ABC-transporter was significantly reduced [82]. Furthermore, under short duration spaceflight missions certain ABC transporter genes in the medically relevant species* Salmonella* sp. and* Candida* sp. were upregulated [83, 84].

Interestingly, we identified many of the g-sensitive genes not only reacting on *μ*g, but also on hypergravity indicating that not only the experimental g-conditions should be taken into account when selecting an appropriate reference gene, but also the accompanying g-conditions prevailing usually before *μ*g is achieved. A detailed differential gene expression analysis of the parabolic flight and sounding rocket flight data sets for g-sensitive genes is currently ongoing.

Genes that proved to be stable over all g-conditions tested, were

- albumin (ALB), a protein comprising about one half of blood serum protein,
- UDP-Gal:betaGlcNAc beta 1,4-galactosyltransferase, polypeptide 6 (B4GALT6), a type II membrane-bound glycoprotein important for glycolipid biosynthesis,
- glyceraldehyde-3-phosphate dehydrogenase (GAPDH), a protein with several distinct functions, for example, the reversible oxidative phosphorylation of glyceraldehyde-3-phosphate,
- hydroxymethylbilane synthase (HMBS), a protein catalyzing the head to tail condensation of four porphobilinogen molecules into the linear hydroxymethylbilane,
- tyrosine 3-monooxygenase/tryptophan 5-monooxygenase activation protein, zeta (YWHAZ), a gene product belonging to the 14-3-3 family of proteins that interacts with IRS1, suggesting a role in regulating insulin sensitivity,
- ATP-binding cassette, subfamily A, member 5 (ABCA5), a membrane-associated protein belonging to the only major ABC subfamily found exclusively in multicellular eukaryotes with unknown function,
- ATP-binding cassette, subfamily A, member 9 (ABCA9), another ABC1 family member induced during monocyte differentiation into macrophages, and
- ATP-binding cassette, subfamily C, member 1 (ABCC1), a member of the MRP subfamily of ABC transporters involved in multidrug resistance and functioning as a multispecific organic anion transporter.

Taken together, the compilation of genes that we present in Table 7 gives an overview about which genes are stably expressed during all investigated gravitational conditions lasting from seconds to minutes and can therefore be considered as suitable reference genes. Furthermore, Table 7 can be regarded as a tool for the community that can be easily adapted to select potential control genes in the design phase of a new, immune cell based, experiment on parabolic flights and sounding rocket flights because it provides valuable information about gene expression levels in *μ*g, as well as in 1.8 g, in-flight 1 g and hardware ground control. Our results also allow for the identification of adaptation mechanisms by comparing short (parabolic flight) and intermediate (sounding rocket) microgravity periods and spot those genes that convert from sensitive into stable and vice versa. Our work should considerably facilitate identification of appropriate reference genes for individual experiments performed during parabolic flight and sounding rocket campaigns with immune cells, especially of the monocyte/macrophage system, in altered gravity.

## Supplementary Material

Supplementary Table 1 summarizes the differential gene expression analysis of the potential reference genes presented in our study. Fluorescence, mean fluorescence and standard deviation values are given for the individual gene probes at different g-conditions measured during the 19th DLR Parabolic flight campaign as well as for the TEXUS-49 sounding rocket mission. Gene expression levels were compared between the different g-conditions for those genes that are represented by at least 3 probes and p-values were calculated. Significant p-values below 0,05 are displayed in red.
Supplementary Table 2 shows the analysis of the ABC transporter genes. Fluorescence, mean fluorescence and standard deviation values are given for the individual gene probes at different g-conditions measured during the 19th DLR Parabolic flight campaign as well as for the TEXUS-49 sounding rocket mission. Gene expression levels were compared between the different gconditions for those genes that are represented by at least 3 probes and p-values were calculated. Significant p-values below 0,05 are displayed in red.
Supplementary Table 3 displays the analyzed tRNA related genes. Fluorescence, mean fluorescence and standard deviation values are given for the individual gene probes at different g-conditions measured during the 19th DLR Parabolic flight campaign as well as for the TEXUS-49 sounding rocket mission. Gene expression levels were compared between the different g-conditions for those genes that are represented by at least 3 probes and p-values were calculated. Significant p-values below 0,05 are displayed in red.

## Figures and Tables

**Table 1:** Gravity conditions 19th DLR PFC and TEXUS-49.

Gravity condition	19th DLR PFC	TEXUS-49
1 g ground controls (hardware; H/W)	H/W	H/W
Microgravity	*μ*g (20 sec)	*μ*g (378 sec)
1 g in-flight control	1 g	—
In-flight baseline (hyper-g phase directly before *μ*g; BL)	1.8 g (20 sec)	BL (1 g—max. 13.5 g; 75 sec)

**Table 2:** List of potential reference genes.

Potential reference gene	Gene symbol	Citation
5s rRNA		[85]
18s rRNA		[86]
*β*-Actin	ACTB	[49, 51]
Albumin	ALB	[49]
*β*-2 microglobin	B2M	[49, 51, 56]
UDP-Gal:bGlcNAcb 1,4-galactosyl-transferase, polypeptide 6	B4GALT6	[86]
Glucose 6-phosphate dehydrogenase	G6PD	[49]
Glyceraldehyde-3-phosphate dehydrogenase	GAPDH	[49, 51, 56, 85, 87, 88]
Glucuronidase, beta	GUSB	[86]
Hydroxymethylbilane synthase (porphobilinogen deaminase)	HMBS	[49, 51, 56]
Hypoxanthine phosphoribosyltransferase 1	HPRT1	[49, 51, 56, 86]
Heat shock protein 90 kDa	HSP90AA1	[86]
Phospholipase A2	PLA2G4A	[49]
RNA polymerase II	POLR2A	[49, 86]
Peptidylprolyl isomerase A (Cyclophilin A)	PPIA	[49, 86]
Ribosomal protein L13	RPL13A	[49, 51, 56, 86]
Acidic ribosomal phosphoprotein P0	RPLP0	[89]
Succinate dehydrogenase complex, subunit A	SDHA	[51, 56, 86]
TATA box binding protein	TBP	[49, 51, 56, 86]
*α*-Tubulin	TUBA1	[49]
Ubiquitin C	UBC	[51, 56]
Tyrosine 3-monooxygenase tryptophan 5-monooxygenase activation protein	YWHAZ	[51, 56, 86]

**Table 3:** Selected potential reference genes (19th DLR PFC).

Gene symbol	Accession number	19th DLR PFC: potential reference genes											
		H/W		1 g		1.8 g		*µ*g		*P* values			
		Mean FI	SD	Mean FI	SD	Mean FI	SD	Mean FI	SD	H/W versus 1 g	1 g versus 1.8 g	1.8 g versus *µ*g	1 g versus *µ*g
ALB	NM_000477	2.13	2.04	40.28	1.22	40.62	0.88	40.10	0.71	0.2627	0.7154	0.4724	0.8383
	BC035969												
	BC034023												

B4GALT6	NM_004775	40.55	7.65	39.30	7.62	43.41	11.38	39.92	7.62	0.8506	0.6342	0.6847	0.9253
	AF069054												
	BC074835												

GAPDH	NM_002046	21,791.23	914.23	21,162.32	1,447.99	19,124.58	1,319.50	20,823.21	2,005.00	0.5652	0.1466	0.2971	0.8250
	BC001601												
	BC009081												

HMBS	NM_000190	2,184.20	973.77	1,612.83	677.49	998.73	443.39	1,226.98	531.59	0.4563	0.2695	0.5994	0.4833
	NM_001024382												
	BC008149												

RPLP0	NM_001002	11,352.34	1,728.26	11,633.77	426.19	11,380.89	2,060.50	12,714.95	837.10	0.7397	0.8004	0.2346	**0.0425**
	BC001127												
	BC008594												
	BC000087												
	BC070194												

TBP	NM_003194	4,158.56	310.25	3,271.43	217.26	2,287.20	161.09	2,776.82	170.76	**0.0192**	**0.0042**	**0.0226**	**0.0390**
	X54993												
	BC109053												

YWHAZ	NM_003406	9,136.31	2,070.39	9,445.22	1,990.27	9,843.30	2,125.25	9,853.06	2,184.44	0.7808	0.7239	0.9934	0.7214
	BC068456												
	BC108281												
	BC101483												
	BC083508												
	BC072426												
	BC003623												

**Table 4:** Selected potential reference genes (TEXUS-49).

Gene symbol	Accession number	TEXUS-49: potential reference genes								
		H/W		BL		*µ*g		*P* values		
		Mean FI	SD	Mean FI	SD	Mean FI	SD	H/W versus BL	BL versus *µ*g	H/W versus *µ*g
ALB	NM_000477	18.02	1.29	17.47	1.18	17.88	1.88	0.6184	0.7708	0.9215
	BC035969									
	BC034023									

B4GALT6	NM_004775	17.15	4.26	17.67	6.05	15.95	3.26	0.9093	0.6933	0.7197
	AF069054									
	BC074835									

GAPDH	NM_002046	23,385.82	1,721.78	24,862.07	2,271.02	25,451.17	1,692.90	0.4238	0.7383	0.2126
	BC001601									
	BC009081									

HMBS	NM_000190	2,921.40	1,084.02	3,087.49	1,094.22	2,568.23	899.65	0.8609	0.5612	0.6872
	NM_001024382									
	BC008149									

RPLP0	NM_001002	8,027.10	1,233.45	9,036.11	1,538.20	7,406.41	2,622.46	0.2871	0.2728	0.6499
	BC001127									
	BC008594									
	BC000087									
	BC070194									

TBP	NM_003194	3,738.25	143.90	4,466.01	257.59	3,517.69	53.87	**0.0215**	**0.0201**	0.1037
	X54993									
	BC109053									

YWHAZ	NM_003406	3,970.64	1,498.10	5,738.74	2,118.20	3,776.01	1,596.71	0.0993	0.0758	0.8180
	BC068456									
	BC108281									
	BC101483									
	BC083508									
	BC072426									
	BC003623									

**Table 5:** Selected ABC transporters (19th DLR PFC).

No.	Gene symbol	Accession number	19th DLR PFC: ABC transporter genes											
			H/W		1 g		1.8 g		*µ*g		*P* values			
			Mean FI	SD	Mean FI	SD	Mean FI	SD	Mean FI	SD	H/W versus 1 g	1 g versus 1.8 g	1.8 g versus *µ*g	1 g versus *µ*g
23	ABCC1	AB209120	3,140.46	751.71	3,309.29	743.99	2,258.45	325.89	2,934.79	496.93	0.7959	0.1193	0.1312	0.5141
		NM_019900												
		NM_004996												

29	ABCC4	BC041560	1,497.37	199.75	1,437.84	279.45	857.05	30.65	1,067.01	91.26	0.7805	0.0676	**0.0459**	0.1380
		AY133678												
		NM_005845												

37	ABCD4	NM_005050	1,719.37	164.62	1,254.13	240.26	765.94	235.75	1,155.89	257.53	**0.0222**	**0.0273**	0.0673	0.5972
		BC012815												
		NM_020326												
		NM_020325												

40	ABCF2	NM_005692	7,510.96	664.99	6,774.22	407.85	6,022.09	525.91	6,525.51	515.17	0.1916	0.1263	0.3019	0.5496
		BC001661												
		AF091073												

47	TAP2	NM_018833	3,365.61	136.24	2,614.83	94.54	2,485.22	190.87	2,027.84	74.61	**0.0023**	0.3711	**0.0394**	**0.0014**
		BC002751												
		AF105151												

**Table 6:** Selected ABC transporters (TEXUS-49).

No.	Gene symbol	Accession number	TEXUS-49: ABC transporter genes								
			H/W		BL		*µ*g		*P* values		
			Mean FI	SD	Mean FI	SD	Mean FI	SD	H/W versus BL	BL versus *µ*g	H/W versus *µ*g
23	ABCC1	AB209120	2,405.02	686.83	3,096.07	634.60	2,020.76	398.49	0.2701	0.0797	0.4598
		NM_019900									
		NM_004996									

29	ABCC4	BC041560	765.34	293.67	1,382.37	294.64	537.87	140.92	0.0620	**0.0228**	0.3164
		AY133678									
		NM_005845									

37	ABCD4	NM_005050	759.92	190.32	843.89	94.39	565.11	138.98	0.4697	**0.0194**	0.1539
		BC012815									
		NM_020326									
		NM_020325									

40	ABCF2	NM_005692	8,382.41	751.10	7,963.43	721.46	6,373.22	573.28	0.5244	**0.0430**	**0.0238**
		BC001661									
		AF091073									

47	TAP2	NM_018833	4,902.84	12.08	4,000.89	35.26	3,430.97	49.27	**0.0001**	**0.0002**	**0.0002**
		BC002751									
		AF105151									

**Table 7:** Overview of g-stable (+) and g-sensitive genes (−).

Gene symbol	Accession number	19th DLR PFC				TEXUS-49		
		(H/W versus 1 g)	(1 g versus 1.8 g)	(1.8 g versus *µ*g)	(1 g versus *µ*g)	(H/W versus BL)	(BL versus *µ*g)	(H/W versus *µ*g)
Potential reference genes								
**ALB**	NM_000477	+	+	+	+	+	+	+
	BC035969							
	BC034023							
**B4GALT6**	NM_004775	+	+	+	+	+	+	+
	AF069054							
	BC074835							
**GAPDH**	NM_002046	+	+	+	+	+	+	+
	BC001601							
	BC009081							
**HMBS**	NM_000190	+	+	+	+	+	+	+
	NM_001024382							
	BC008149							
RPLP0	NM_001002	+	+	+	−	+	+	+
	BC001127							
	BC008594							
	BC000087							
	BC070194							
TBP	NM_003194	−	−	−	−	−	−	+
	X54993							
	BC109053							
**YWHAZ**	NM_003406	+	+	+	+	+	+	+
	BC068456							
	BC108281							
	BC101483							
	BC083508							
	BC072426							
	BC003623							

ABC transporter genes								
**ABCA5**	NM_018672	+	+	+	+	+	+	+
	AJ275973							
	AY028897							
**ABCA9**	NM_080283	+	+	+	+	+	+	+
	BC062472							
	NM_172386							
**ABCC1**	AB209120	+	+	+	+	+	+	+
	NM_019900							
	NM_004996							
ABCC4	BC041560	+	+	−	+	+	−	+
	AY133678							
	NM_005845							
ABCC12	AK127951	−	+	+	+	−	−	+
	NM_145187							
	NM_033226							
	BC036378							
ABCD4	NM_005050	−	−	+	+	+	−	+
	BC012815							
	NM_020326							
	NM_020325							
ABCF2	NM_005692	+	+	+	+	+	−	−
	BC001661							
	AF091073							
TAP2	NM_018833	−	+	−	−	−	−	−
	BC002751							
	AF105151							

## Abbreviations

BL: Baseline
CEV: Centre d'essai en vol
CV: Coefficient of variation
ESA: European Space Agency
FBS: Fetal bovine serum
FDR: False discovery rate
DLR: German Aerospace Center
H/W: Hardware ground controls
HKG: Housekeeping genes
hyper-g: Hypergravity
IL: Interleukin
ISS: International Space Station
LPS: Lipopolysaccharide
*μ*g: Microgravity
MMS: Monocyte-macrophage system
PFC: Parabolic flight campaign
qPCR: Quantitative real-time PCR
RIN: RNA integrity number
RPM: Random positioning machine
ROS: Reactive oxygen species
RWV: Rotating wall vessel
SD: Standard deviation
TNF-*α*: Tumor necrosis factor-alpha.
